# Bidirectional regulation of synaptic transmission by BRAG1/*IQSEC2* and its requirement in long-term depression

**DOI:** 10.1038/ncomms11080

**Published:** 2016-03-24

**Authors:** Joshua C. Brown, Amber Petersen, Ling Zhong, Miranda L. Himelright, Jessica A. Murphy, Randall S. Walikonis, Nashaat Z. Gerges

**Affiliations:** 1 Department of Cell Biology, Neurobiology and Anatomy, The Medical College of Wisconsin, Milwaukee, Wisconsin 53132 USA; 2 Department of Physiology and Neurobiology, University of Connecticut, Storrs, Connecticut 06269 USA

## Abstract

Dysfunction of the proteins regulating synaptic function can cause synaptic plasticity imbalance that underlies neurological disorders such as intellectual disability. A study found that four distinct mutations within BRAG1, an Arf-GEF synaptic protein, each led to X-chromosome-linked intellectual disability (XLID). Although the physiological functions of BRAG1 are poorly understood, each of these mutations reduces BRAG1's Arf-GEF activity. Here we show that BRAG1 is required for the activity-dependent removal of AMPA receptors in rat hippocampal pyramidal neurons. Moreover, we show that BRAG1 bidirectionally regulates synaptic transmission. On one hand, BRAG1 is required for the maintenance of synaptic transmission. On the other hand, BRAG1 expression enhances synaptic transmission, independently of BRAG1 Arf-GEF activity or neuronal activity, but dependently on its C-terminus interactions. This study demonstrates a dual role of BRAG1 in synaptic function and highlights the functional relevance of reduced BRAG1 Arf-GEF activity as seen in the XLID-associated human mutations.

## Abstract (web summary)

BRAG1 mutations are linked to synaptic deficits and X-chromosome linked intellectual disability. Here, the authors show that BRAG1 mediates activity-dependent removal of synaptic AMPA receptors via Arf-GEF activity and PDZ interactions, and is required for maintaining AMPAR-mediated synaptic transmission.

## Graphical abstract

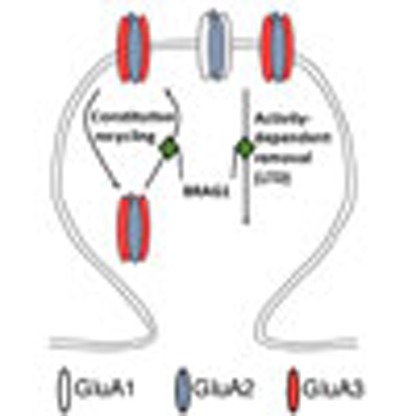

Individuals with intellectual disability have impaired cognitive function and deficits in adaptive behaviour beginning during development. Intellectual disability occurs frequently in the general population, affecting some 2–3% of individuals. However, the cause of intellectual disability is unknown in up to 60% of the cases. In many cases, the genetic cause of intellectual disability can be linked to defects in the X chromosome. Shoubridge *et al*. recently discovered four mutations in the gene *IQSEC2* that cause nonsyndromic X-chromosome-linked intellectual disability (XLID)^1^. *IQSEC2* encodes a protein called BRAG1 or IQSEC2, a highly abundant protein within the postsynaptic density (PSD) of glutamatergic synapses. BRAG1 functions as a guanine nucleotide exchange factor (GEF) for ADP-ribosylation factors (Arfs) through its Sec7 domain. All four BRAG1 XLID mutations result in decreased Arf-GEF activity^1^. However, it is not well understood how decreased enzymatic activity influences synaptic function and plasticity.

BRAG1, a member of the Brefeldin A-resistant Arf-GEF family, is a multi-domain protein containing an IQ-like motif, a Sec7 domain and a C-terminal sequence that can bind to type I PDZ domains. BRAG1 was originally identified in the PSD fraction by mass spectrometry^2^,^3^,^4^,^5^. BRAG1 is one of the most abundant proteins in the PSD, ranking higher than NMDA (*N*-methyl-D-aspartate) receptors or AMPA (α-amino-3-hydroxy-5-methyl-4-isoxazole propionic acid) receptor subunits GluA1 and GluA3 (ref. 6). This suggests a pivotal importance in synaptic function.

Synaptic function is dependent on the targeting and delivery of various proteins to the postsynaptic membrane. One such important protein is the AMPA-type glutamate receptor (AMPAR). While AMPARs mediate most excitatory synaptic transmission in the brain, they are highly dynamic components of excitatory synapses. AMPARs undergo two distinct trafficking modes, regulated and constitutive. The regulated, activity-dependent addition and removal of AMPARs lead to long-term potentiation (LTP) and depression (LTD), respectively^7^,^8^,^9^,^10^,^11^,^12^,^13^,^14^,^15^,^16^,^17^. In addition to this regulated trafficking, AMPARs constitutively cycle in and out of synapses in an activity-independent manner^18^,^19^,^20^,^21^,^22^,^23^. Over the past decade, we have learned a great deal, on one hand, about neurotransmitter receptor binding and anchoring proteins (for example, membrane-associated guanylate kinases, MAGUKs)^24^,^25^,^26^,^27^,^28^,^29^,^30^,^31^,^32^, and on the other, about membrane trafficking proteins (for example, small GTPases)^18^,^33^,^34^,^35^. Nonetheless, the mechanistic links between these two types of elements are not well understood.

Here we have explored a potential role for BRAG1, a regulator of a membrane trafficking protein Arf6 (due to its Arf-GEF activity) that can also bind to PDZ domain postsynaptic anchoring proteins (due to its PDZ-binding motif), in the regulation of AMPAR trafficking. Using a combination of molecular biology, biochemistry, electrophysiology and fluorescence imaging, we have found that the Arf-GEF activity of BRAG1 is necessary for the regulated removal of AMPARs, which is essential to maintain NMDAR-mediated LTD, and thus the balance of synaptic plasticity. We also found that BRAG1 plays a bidirectional role in the regulation of synaptic transmission. On one hand, BRAG1 is required for the maintenance of synaptic AMPARs. On the other hand, BRAG1 has a rate-limiting role in the insertion of AMPA receptors that is independent of its enzymatic function.

## Results

### BRAG1 enhances AMPA receptor-mediated responses

**Figure 1:**
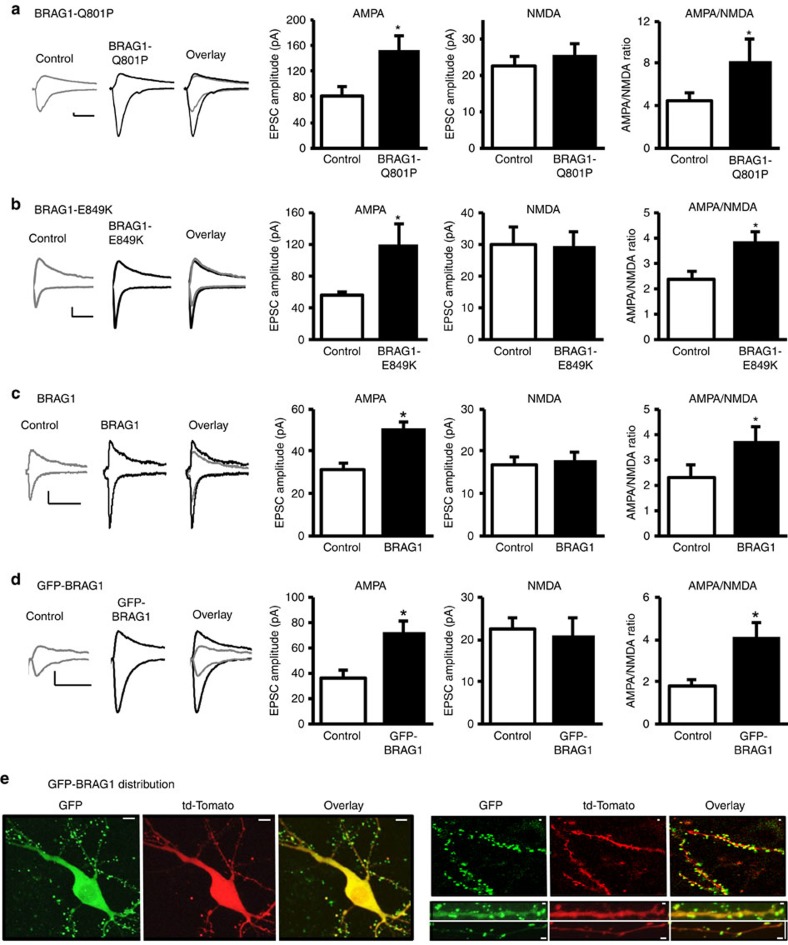
BRAG1 enhances AMPA receptor-mediated responses independently of its enzymatic activity. (**a**–**d**) Left: sample traces of AMPAR- and NMDAR-mediated synaptic responses recorded at −60 and+40 mV (the amplitude at 60 ms latency, after AMPAR EPSCs are decayed), respectively. Scale bars, 20 pA, 20 ms. Data represent averaged evoked EPSCs recorded for AMPAR (left graphs), NMDAR (middle graphs) and AMPA/NMDA ratio (right graphs) simultaneously from pairs of untransfected (control) CA1 neurons and neurons transfected with BRAG1-Q801P (**a**; AMPA: *n*=11, *P*=0.0050; NMDA: *n*=8, *P*=0.17; AMPA/NMDA: *n*=8, *P*=0.019), BRAG1-E849K (**b**; AMPA: *n*=15, *P*=0.0079; NMDA: *n*=11, *P*=0.55; AMPA/NMDA: *n*=11, *P*=0.0063), BRAG1 (**c**; AMPA: *n*=18, *P*=0.00034; NMDA: *n*=18, *P*=0.40; AMPA/NMDA: *n*=14, *P*=0.0019) or GFP-tagged BRAG1 (**d**; AMPA: *n*=10, *P*=0.010; NMDA: *n*=9, *P*=0.92; AMPA/NMDA: *n*=8, *P*=0.013). * Indicates significance (*P*≤0.05). (**e**) Confocal images of GFP-BRAG1 co-expressed with td-Tomato in hippocampal CA1 neurons (left; scale bars, 10 μm) and their dendritic distribution (right; scale bars, 1 μm) showing preferential localization of BRAG1 at dendritic spines.

The four identified mutations in BRAG1 associated with XLID result in decreased Arf-GEF activity^1^. To understand the effect of intellectual disability-associated mutations on synaptic function, we expressed the human BRAG1-Q801P mutant, which causes more severe intellectual disability than the other mutants, in organotypic hippocampal slices. The effect of BRAG1-Q801P on synaptic transmission was evaluated by simultaneous double whole-cell recordings from pairs of nearby transfected and untransfected neurons under voltage-clamp configuration. Surprisingly, overnight expression of BRAG1-Q801P significantly increased AMPAR-mediated responses, without affecting NMDAR-mediated responses (Fig. 1a). To further investigate the role of BRAG1 enzymatic activity in synaptic function, we expressed BRAG1-E849K, a dominant-negative mutant that lacks Arf-GEF enzymatic activity^1^,^36^, in organotypic slices overnight. Similar to BRAG1-Q801P, BRAG1-E849K also enhanced AMPA receptor-mediated responses (Fig. 1b). These data may suggest that decreased BRAG1 Arf-GEF enzymatic activity results in the enhancement of AMPAR-mediated responses. Alternatively, this enhancement could result from an enzymatic-independent function of BRAG1. To this end, we tested the effect of overexpressing wild-type BRAG1 (BRAG1) on synaptic transmission. As shown in Fig. 1c, BRAG1 expression also increased AMPAR-mediated responses, indicating that the increase in synaptic transmission is unrelated to its enzymatic activity. These results were further validated by the expression of green fluorescent protein (GFP)-tagged BRAG1, which also increases AMPAR-mediated responses (Fig. 1d). GFP-BRAG1 concentrates at dendritic spines (Fig. 1e), comparable to endogenous BRAG1, as shown previously for GFP-tagged BRAG1 (refs 4, 37). To note, neither wild-type BRAG1 nor any of these mutants affected passive membrane properties of the transfected cells, such as input resistance and holding current (Supplementary Fig. 1).

The effects of BRAG1 expression are specific to AMPA receptors as neither NMDA receptor-mediated responses (Fig. 1) nor GABA (γ-aminobutyric acid) receptor-mediated responses were affected as a result of its expression (Supplementary Fig. 2). There was no correlation between the age of the slice culture at the time of recording and the BRAG1-mediated enhancement of synaptic transmission within the time frame used (Supplementary Fig. 3).

Although BRAG1 is expressed in postsynaptic CA1 neurons, we also tested whether BRAG1 indirectly interferes with presynaptic mechanisms. We measured paired pulse facilitation (PPF), a form of short-term synaptic plasticity and an indicator of presynaptic function, in both control and BRAG1-expressing neurons. PPF is unaltered by BRAG1 expression (Supplementary Fig. 4), which confirms that BRAG1-mediated enhancement of synaptic transmission is not because of a presynaptic effect.

### BRAG1 C terminus is required for synaptic enhancement

**Figure 2:**
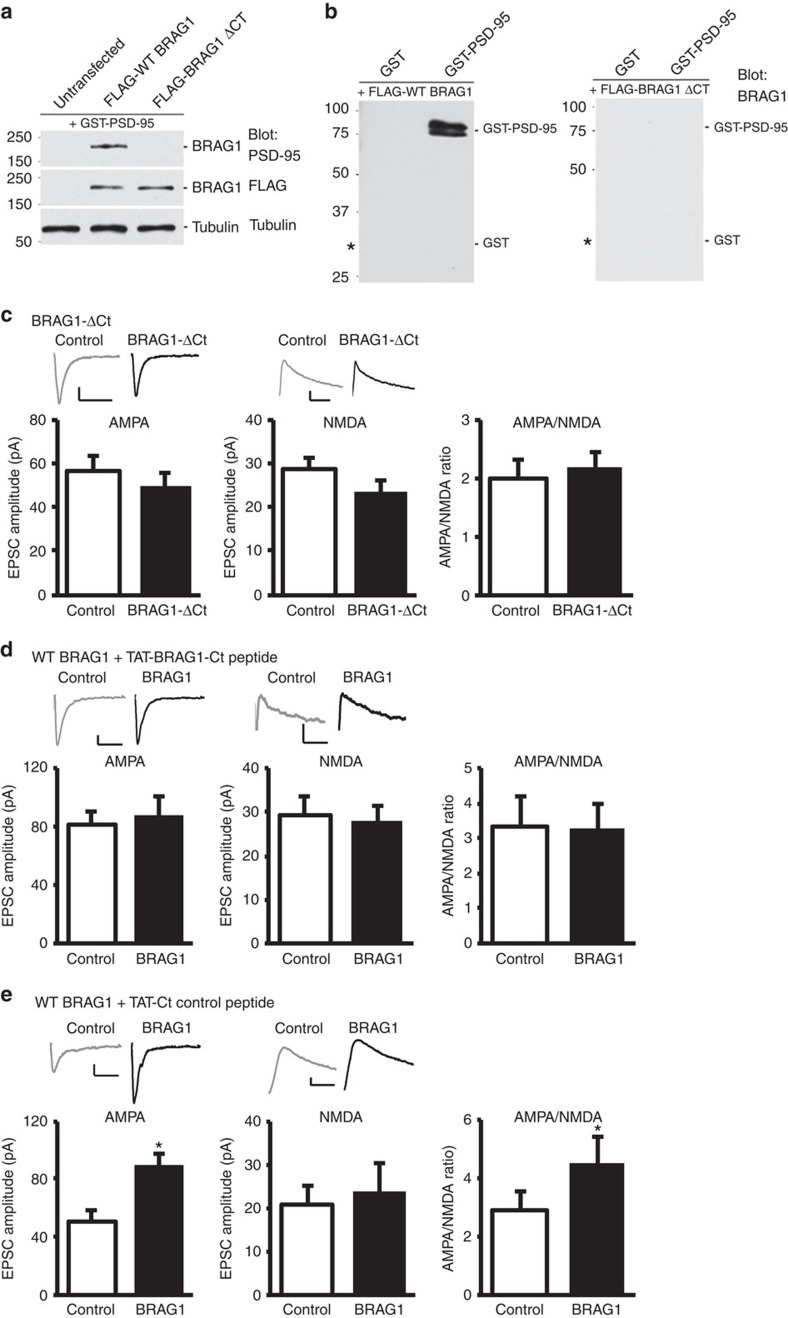
BRAG1-mediated enhancement of synaptic transmission depends on its C-terminus interactions. (**a**,**b**) BRAG1's C terminus interacts with PSD-95 *in vitro*. (**a**) Protein overlay assay demonstrating the interaction between WT BRAG1 and recombinant PSD-95. Top: HEK293 cell lysates expressing FLAG-WT BRAG1, FLAG-BRAG1 C-terminal deletion mutant (FLAG-BRAG1ΔCt) or no plasmid (untransfected) were run on the gel, and the membrane was overlaid with recombinant GST-PSD-95, and stained with antiserum UCT80 against PSD-95. Middle and bottom: the blot was also probed with anti-FLAG to detect total BRAG1 in the lysates and with anti-tubulin as a loading control. (**b**) Left: GST-PSD-95 and GST were separated by SDS–polyacrylamide gel electrophoresis and transferred to a nitrocellulose membrane, which was overlaid with a HEK293 cell lysate overexpressing FLAG-WT BRAG1 and stained with antiserum C3 against BRAG1. BRAG1 binds to GST-PSD-95, but not GST (indicated by the *). Right: protein overlay assay with FLAG-BRAG1ΔCt. GST-PSD-95 and GST were run on the gel, and the membrane was overlaid with a HEK293 cell lysate overexpressing FLAG-BRAG1 ΔCt and stained with anti-FLAG to detect BRAG1. WT BRAG1 but not BRAG1ΔCt binds to GST-PSD-95. Molecular weight markers in kDa are shown on the left. (**c**–**e**) Insets: sample traces of AMPAR- and NMDAR-mediated synaptic responses recorded at −60 (left) and +40 mV (right), respectively. Scale bars, 20 pA, 20 ms. Data represent averaged evoked EPSCs recorded for AMPA (left graphs), NMDA (centre graphs) and AMPA/NMDA ratios (right graphs) simultaneously from pairs of untransfected (control) CA1 neurons and neurons transfected with BRAG1-ΔCt (**c**; AMPA: *n*=18, *P*=0.24; NMDA: *n*=18, *P*=0.24; AMPA/NMDA: *n*=15, *P*=0.12) or BRAG1 (**d**,**e**). In **d** and **e**, shortly after BRAG1 transfection, TAT-BRAG1-Ct peptide (**d**; AMPA: *n*=10, *P*=0.67; NMDA: *n*=7, *P*=0.25; AMPA/NMDA: *n*=7, *P*=0.97) or TAT-Ct control peptide (**e**; AMPA: *n*=7, *P*=0.031; NMDA: *n*=8, *P*=0.42, AMPA/NMDA: *n*=7, *P*=0.047) was added to the culture media. * Indicates significance (*P*≤0.05).

The increase in AMPAR-mediated responses resulting from BRAG1 expression reveals a surprising role for BRAG1 in synaptic transmission that is independent of its enzymatic activity. The last four amino acids of the C terminus of BRAG1 (STVV) conform to the consensus sequence for binding to type I PDZ domains. Proteins containing PDZ domains often participate in trafficking of synaptic proteins and organization of proteins involved in synaptic transmission. To determine whether binding of BRAG1 to PDZ domains is required for the BRAG1-mediated increase in AMPAR-mediated responses, we expressed a truncated form of BRAG1 that lacks the last nine amino acids (BRAG1-ΔCt), making it unable to bind to PDZ domain proteins (Fig. 2a,b). Images have been cropped for presentation. Full-size images are presented in Supplementary Fig. 5. As shown in Fig. 2c, BRAG1-ΔCt failed to enhance AMPAR-mediated responses, indicating a crucial role for PDZ interactions in BRAG1-mediated enhancement of AMPAR-mediated response. To further validate this finding, we used a peptide identical to the last nine amino acids of BRAG1 to competitively interfere with BRAG1 binding to PDZ proteins. The peptide was tagged with TAT (transactivator of transcription), an approach used successfully to deliver peptides into neurons^38^,^39^,^40^,^41^,^42^. The identity and purity of the peptides were verified by mass spectral analysis (Supplementary Fig. 6). To directly test whether the BRAG1-induced increase in AMPAR-mediated responses requires a BRAG1–PDZ interaction, we incubated organotypic hippocampal slices with TAT-BRAG1-Ct peptide immediately after the transfection of BRAG1. As shown in Fig. 2d, TAT-BRAG1-Ct peptide blocked the BRAG1-mediated increase in AMPAR excitatory postsynaptic currents (EPSCs), indicating that the BRAG1–PDZ-binding sequence is required for such an effect. Importantly, a control peptide, in which the BRAG1 C-terminal STVV sequence was replaced with C-terminal SAVA to render the peptide incapable of binding to corresponding PDZ domains, did not interfere with the BRAG1-mediated enhancement (Fig. 2e).

**Figure 3:**
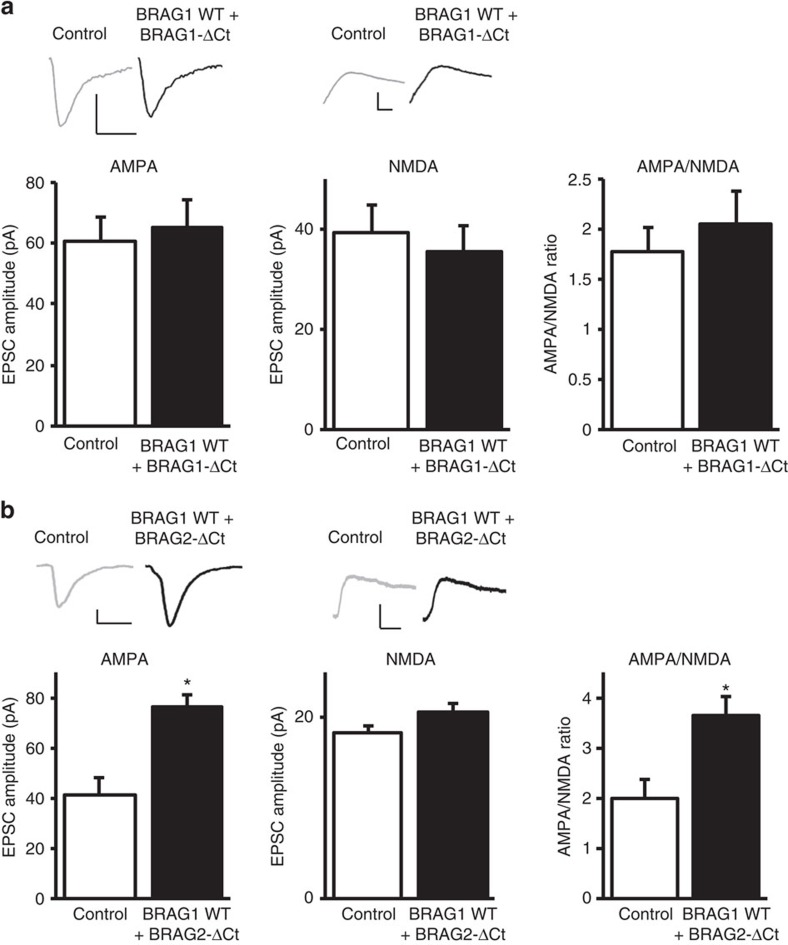
Expression of BRAG1, but not BRAG2, lacking the PDZ-binding sequence interfere with BRAG1-mediated enhancement of synaptic transmission. (**a**,**b**) Insets: sample traces of AMPAR- and NMDAR-mediated responses recorded at −60 and +40 mv, respectively. Scale bars, 20 pA, 20 ms. Data represent averaged evoked EPSCs recorded for AMPA (left graphs), NMDA (centre graphs) and AMPA/NMDA ratios (right graphs) simultaneously from pairs of untransfected (control) CA1 neurons and neurons transfected with BRAG1+BRAG1-ΔCt (**a**; AMPA: *n*=11, *P*=0.65; NMDA: *n*=11, *P*=0.34; AMPA/NMDA: *n*=11, *P*=0.08) or BRAG1+BRAG2-ΔCt (**b**; AMPA: *n*=8, *P*=0.0063; NMDA: *n*=8, *P*=0.28; AMPA/NMDA: *n*=8, *P*=0.05). * Indicates significance (*P*≤0.05).

To further analyse the role of the PDZ-binding sequence in the BRAG1-mediated increase in AMPAR-mediated responses, we co-expressed BRAG1 with BRAG1-ΔCt. The expression of the BRAG1 mutant that lacks the PDZ-binding sequence blocked BRAG1-mediated increase in AMPAR-mediated responses (Fig. 3a). Since both BRAG1 and BRAG2 are expressed in neurons and they share a similar PDZ-binding sequence, we wished to test whether a BRAG2 lacking the PDZ-binding sequence would interfere with the BRAG1-mediated increase in AMPAR-mediated responses. As shown in Fig. 3b, BRAG2 lacking the PDZ-binding sequence did not interfere with the ability of BRAG1 to enhance AMPAR-mediated responses. Expression of BRAG2 lacking the PDZ-binding sequence alone had no effect on synaptic transmission (Supplementary Fig. 7). Collectively, these data strongly suggest that the observed BRAG1-mediated enhancement of AMPAR-mediated transmission requires BRAG1's PDZ-binding sequence.

### BRAG1 enhances synaptic strength independently of activity

**Figure 4:**
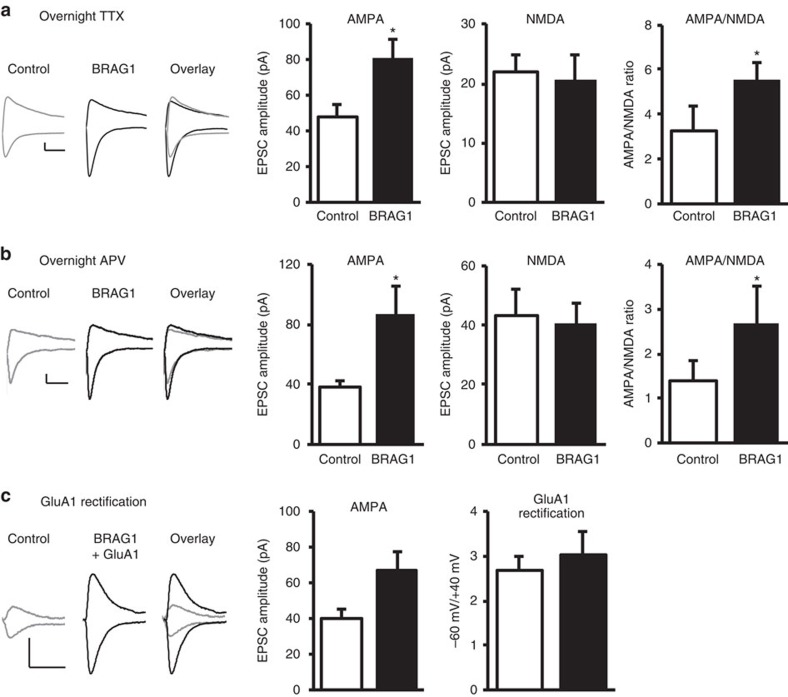
BRAG1 enhances synaptic strength independently of synaptic activity or GluA1 insertion. (**a**,**b**) Insets: sample traces of AMPAR- and NMDAR-mediated synaptic responses recorded at −60 (left) and +40 mV (right), respectively. Scale bars, 20 pA, 20 ms. Data represent averaged evoked EPSCs recorded for AMPA (left graphs), NMDA (middle graphs) and AMPA/NMDA ratios (right graphs) simultaneously from pairs of nearby untransfected and BRAG1-transfected CA1 neurons treated overnight with TTX (**a**; AMPA: *n*=8, *P*=0.013; NMDA: *n*=8, *P*=0.51; AMPA/NMDA: *n*=7, *P*=0.020) or APV (**b**; AMPA: *n*=9, *P*=0.023; NMDA: *n*=6, *P*=0.64; AMPA/NMDA: *n*=6, *P*=0.031). (**c**) Insets: sample traces of evoked AMPAR-mediated synaptic responses recorded at −60 and +40 mV from control cells or cells co-transfected with BRAG1 and GluA1. Scale bars, 20 pA, 20 ms. Left graph shows AMPAR-mediated responses recorded at −60 mV (*n*=14, *P*=0.027). Right graph shows the rectification index, which was calculated as the ratio of the amplitude of AMPAR-mediated responses at −60 mV to that at +40 mV (*n*=13, *P*=0.39). * Indicates significance (*P*≤0.05).

AMPAR subunits are subject to differential regulation and trafficking^43^. GluA1/2 receptors are driven into the synapse in an activity-dependent manner, while GluA2/3 receptors undergo constitutive recycling^19^,^43^,^44^. To test whether BRAG1-mediated potentiation of synaptic transmission is dependent on spontaneous activity, we performed double whole-cell recordings from slices in which spontaneous activity was blocked with the sodium channel blocker tetrodotoxin (TTX, 1 μM) during BRAG1 expression. Under these conditions, BRAG1 was still able to potentiate AMPAR-mediated responses (Fig. 4a), indicating that activity is not required for BRAG1-induced potentiation.

To determine whether BRAG1-mediated potentiation is dependent on NMDAR activation, simultaneous whole-cell double recordings were obtained from transfected and untransfected neurons to which DL-2-amino-5-phosphonopentanoate (APV) was added to block NMDARs during BRAG1 expression, but not during recording. Once again, BRAG1 was able to potentiate synaptic transmission (Fig. 4b), indicating that such potentiation is independent of NMDAR activation.

Since GluA1 insertion requires activation of NMDARs, these data strongly suggest that BRAG1-mediated enhancement of AMPAR-mediated responses is not due to the insertion of GluA1-containing AMPARs. To directly test whether BRAG1-mediated enhancement is due to the insertion of GluA1-containing AMPARs into the synapse, we co-transfected CA1 neurons with GFP-GluA1 and BRAG1, and performed simultaneous whole-cell double recordings. Delivery of GFP-GluA1 receptors to synapses was monitored using the inward rectification properties of the homomeric recombinant receptor (electrophysiological tagging)^45^,^46^,^47^. Synaptic delivery was then quantified as an increase in the ratio of the evoked postsynaptic current at −60 mV relative to the current at +40 mV (rectification index=*I*_−60_/*I*_+40_). As shown in Fig. 4c, while BRAG1 significantly increased AMPAR-mediated responses, it did not change the rectification index; indicating that the increase in AMPAR-mediated responses is not due to the synaptic delivery of homomeric GluA1. It remains unclear, however, how endogenous synaptic receptor complexes are affected.

### BRAG1 increases the synaptic recycling pool of AMPARs

**Figure 5:**
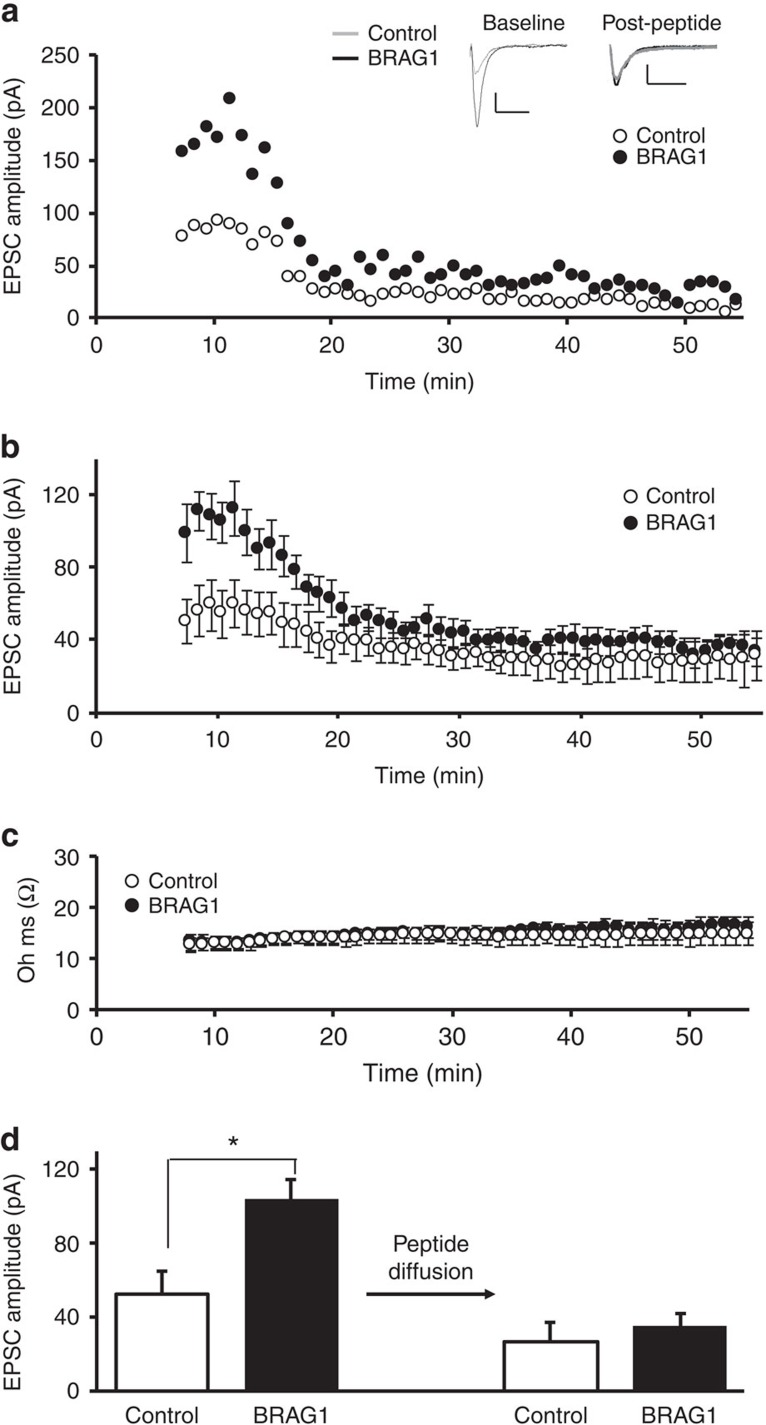
BRAG1 increases the synaptic pool of AMPA receptors that constitutively recycle. (**a**) Graph of representative time-course experiment of whole-cell double patch-clamp recordings from a control neuron and a neuron transfected with BRAG1. In these experiments, patch pipettes contain 10 μM pep2m in the internal solution. Insets: traces of AMPAR-mediated responses of the representative experiment at baseline (thin line, left) and at the new steady state following pep2m infusion (thick line, right). Scale bars, 20 pA, 20 ms. (**b**) Averaged EPSC values from adjacent transfected and control cells. Time zero indicates the time at which neurons were patch clamped at −60 mV. Note that immediately following patch clamping, BRAG1-transfected neurons have higher AMPAR-mediated responses. Pep2m caused rundown in BRAG1-transfected neurons to a higher degree than in control ones. (**c**) Series resistance values corresponding to EPSC values over time (control versus BRAG1, 0–55 min, *n*=8, *P*=0.51). (**d**) Quantification of average EPSC values of baseline (up to 12 min, *n*=8, *P*<0.05) and steady state following peptide infusion (35–55 min, *n*=8, *P*=0.56). * Indicates significance (*P*≤0.05).

GluA2/3 receptors, unlike GluA1/2 receptors, undergo continuous recycling in and out of synapses. This synaptic recycling of GluA2/3 receptors was originally inferred from intracellular peptide infusion experiments, in which AMPAR-mediated synaptic transmission rapidly ran down when a specific peptide competing with the interaction between GluA2 and NSF (*N*-ethylmaleimide-sensitive fusion protein) was loaded into the recorded cell^19^,^21^. We used the same approach to determine whether BRAG1-mediated enhancement of AMPAR-mediated responses is due to an increase in synaptic GluA2/3 receptors. We obtained whole-cell double recordings from pairs of nearby BRAG1-transfected and untransfected control neurons. A peptide that blocks GluA2/NSF interactions (pep2m) was loaded into recording micropipettes and infused into both cells. Recordings obtained immediately after patching the cells (before the peptide exerts its effect) showed an enhancement of EPSPs in BRAG1 cells, similar to our prior data, as we would expect (Fig. 5a). Pep2m infusion caused a gradual decrease in synaptic transmission in control cells, with nearly 50% reduction in amplitude. Interestingly, pep2m caused a greater reduction in EPSPs in BRAG1 cells. AMPAR-mediated responses at the plateau (35–55 min post patching) were indistinguishable in control and BRAG1-expressing cells. Given that BRAG1 cells had a near twofold enhancement in transmission over control cells at baseline, and that peptide infusion ran both BRAG1 and control neurons down to a similar level, these data suggest that the BRAG1 enhancement of AMPAR transmission is mediated by the increased expression of the recycling pool of synaptic GluA2/3 receptors.

### BRAG1 increases surface GluA2 but not GluA1

**Figure 6:**
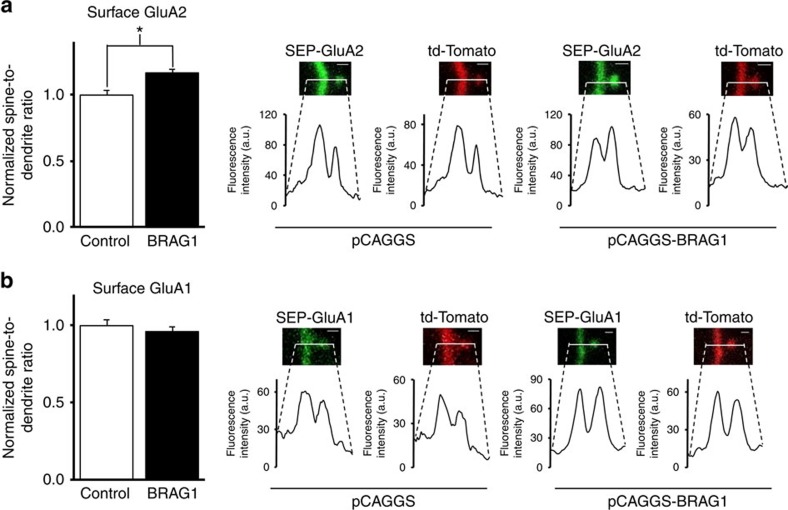
BRAG1 increases surface GluA2 but not GluA1. (**a**) Left: Data represent the average normalized spine-to-dendrite ratios of SEP-GluA2 in the presence and absence of BRAG1. Right: representative confocal images of SEP-GluA2 and td-Tomato co-expressed with either pCAGGS (left, *n*=114) or pCAGGS-BRAG1 (right, *n*=73). (**b**) Left: data represent the average normalized spine-to-dendrite ratios of SEP-GluA1 in the presence and absence of BRAG1. Right: representative confocal images of SEP-GluA1and td-Tomato co-expressed with either pCAGGS (left, *n*=102) or pCAGGS-BRAG1 (right, *n*=102). * Indicates significance (*P*≤0.05). Scale bars, 1 μm.

To directly test whether BRAG1 changes AMPAR surface expression, we monitored the expression of surface AMPAR using the super ecliptic pHluorin GluA2 (SEP-GluA2) or SEP-GluA1 in the presence or absence of BRAG1. As shown in Fig. 6, BRAG1 specifically increases the surface expression of GluA2 and not GluA1. Taken together, these data indicate that BRAG1 mediates increased expression of surface GluA2.

### BRAG1 is required for the synaptic transmission

To further understand the role of BRAG1 in synaptic function, we wanted to investigate the effects of decreasing endogenous BRAG1 on synaptic transmission. To this end, we used a knockdown approach to acutely reduce BRAG1 levels and tested the effects on basal synaptic transmission.

We designed three unique 19-nucleotide sequences for BRAG1 and used the pSuper RNA interference (RNAi) system, which uses a mammalian expression vector that directs intracellular synthesis of small-interfering RNA (siRNA)-like transcripts. These siRNAs significantly downregulate the expression of endogenous BRAG1 in hippocampal neurons (Fig. 5a–c). To test whether acutely knocking down BRAG1 alters synaptic transmission, we carried out simultaneous whole-cell double recordings from neurons co-expressing BRAG1-siRNA and GFP, and untransfected neurons.

**Figure 7:**
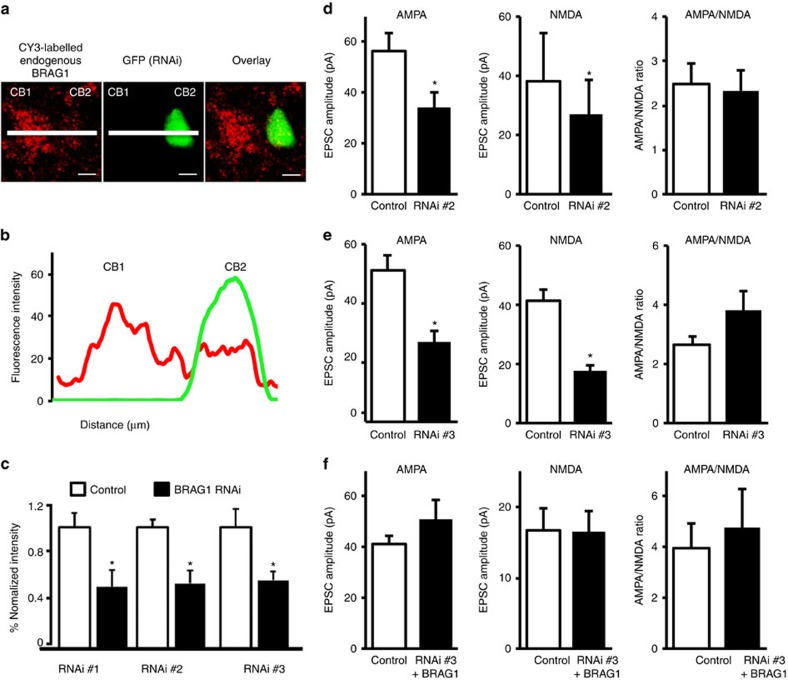
BRAG1 is required for the maintenance of synaptic transmission. (**a**) Representative confocal images from organotypic hippocampal slices transfected with pSuper plasmid containing BRAG1-siRNA and expressing GFP (to visualize the transfected cells) and immunostained 2 days later for BRAG1 (red signal) under permeabilized conditions. Note, the cell transfected with the BRAG1-siRNA (green cell) has low levels of endogenous BRAG1 (red signal). Scale bars, 10 μm. (**b**) Representative line plot analysis of BRAG1 (red signal) and GFP (green signal). CB1 is a cell body of non-transfected neuron; CB2 is that of a transfected neuron. (**c**) Normalized intensity from the line plots for endogenous BRAG1 for the three different RNAi used; RNAi significantly (*P*≤0.05) decreased BRAG1 levels. RNAi #1: *n*=14, *P*=0.0112; RNAi #2: *n*=7, *P*=0.0132; RNAi #3: *n*=10, *P*=0.0417. (**d**,**e**) Simultaneous whole-cell double recordings from nearby pairs of untransfected (control) neurons and those transfected with either BRAG1-siRNA#2 (**d**) or BRAG1-siRNA#3 (**e**). Left graph: comparisons of evoked AMPAR-mediated responses. Right graph: simultaneous recordings of evoked NMDAR-mediated responses (*P*=0.21). (**f**) RNAi #3 was co-expressed with RNAi-resistant WT BRAG1 to restore BRAG1 levels. Data represent average AMPAR EPSCs (left; *n*=10, *P*=0.15), NMDAR EPSCs (middle; *n*=8, *P*=0.51) and AMPA/NMDA ratios (right; *n*=8, *P*=0.60). * Indicates significance (*P*≤0.05).

As shown in Fig. 7d,e, two different BRAG1-siRNA significantly reduced synaptic transmission. Importantly, neurons co-expressing BRAG1-siRNA with GFP-BRAG1 showed synaptic transmission comparable to that of untransfected control neurons, ruling out off-target siRNA effects. These data demonstrate that the reduction of BRAG1 results in a reduction of synaptic transmission. Together with the data showing that overexpression of BRAG1 enhances synaptic transmission, these data highlight the role of BRAG1 in the bidirectional regulation of synaptic transmission.

### BRAG1 C terminus is required for synaptic transmission

**Figure 8:**
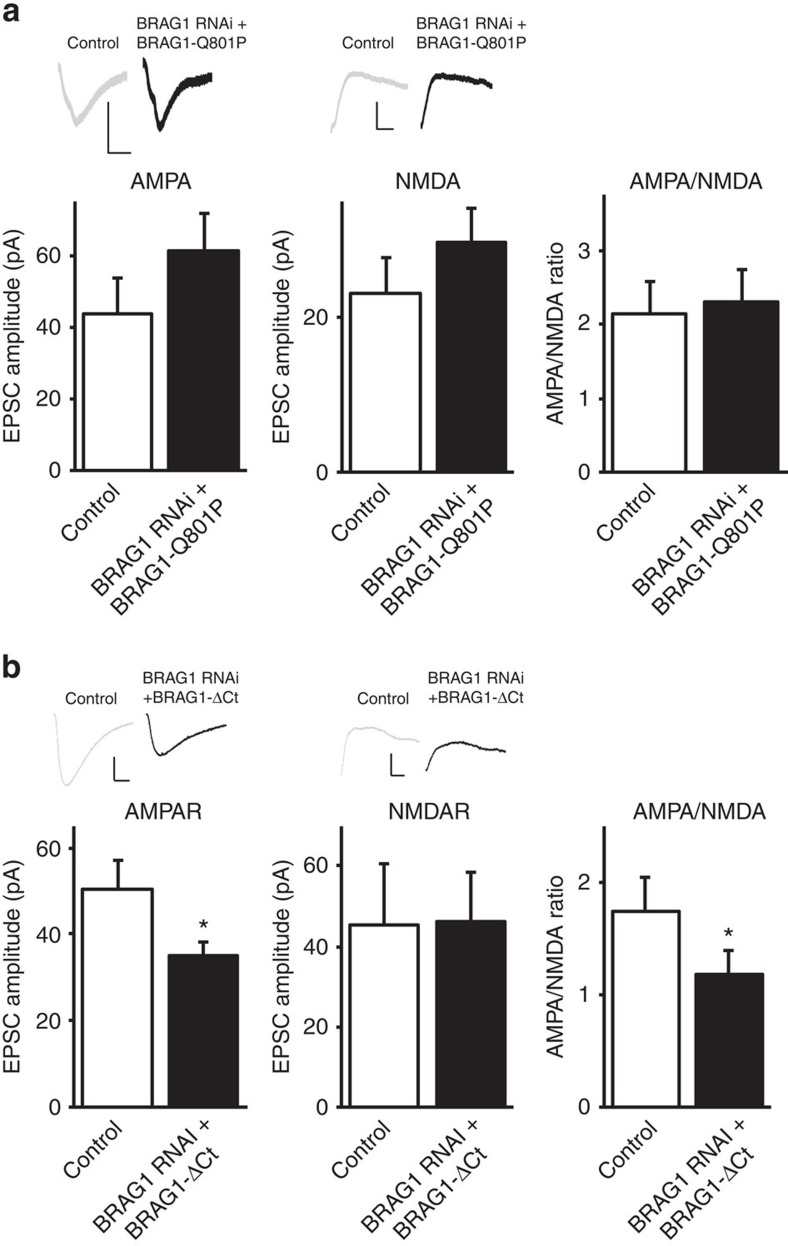
BRAG1–PDZ-binding sequence, but not its Arf-GEF enzymatic activity, is required for the maintenance of synaptic transmission. (**a**,**b**) Left: sample traces of AMPAR- and NMDAR-mediated responses recorded at −60 and +40 mv, respectively. Scale bars, 20 pA, 20 ms. Data represent averaged evoked EPSCs recorded for AMPA (left graphs), NMDA (centre graphs) and AMPA/NMDA ratios (right graphs) simultaneously from pairs of untransfected (control) CA1 neurons and neurons transfected with BRAG1 RNAi+BRAG1-Q801P (**a**) or BRAG1 RNAi+BRAG1-ΔCt (**b**). * Indicates significance (*P*≤0.05).

To further analyse the roles of the PDZ-binding sequence and the Arf-GEF enzymatic activity of BRAG1 in synaptic transmission, we have used a knockdown and replacement strategy^48^. As shown in Fig. 8a, BRAG1-Q801P was able to rescue the RNAi-mediated depression of synaptic transmission, indicating that the BRAG1's enzymatic activity is not required for the maintenance of synaptic transmission. On the other hand, BRAG1-ΔCt was not able to rescue the RNAi-mediated depression of AMPAR-mediated synaptic transmission, further supporting a crucial role of the PDZ-binding sequence of BRAG1 in its role in synaptic transmission (Fig. 8b). Interestingly, BRAG1-ΔCt did rescue the RNAi-mediated depression of NMDAR-mediated responses, suggesting an independent role of BRAG1 in NMDAR function.

### BRAG1 is not required for LTP

**Figure 9:**
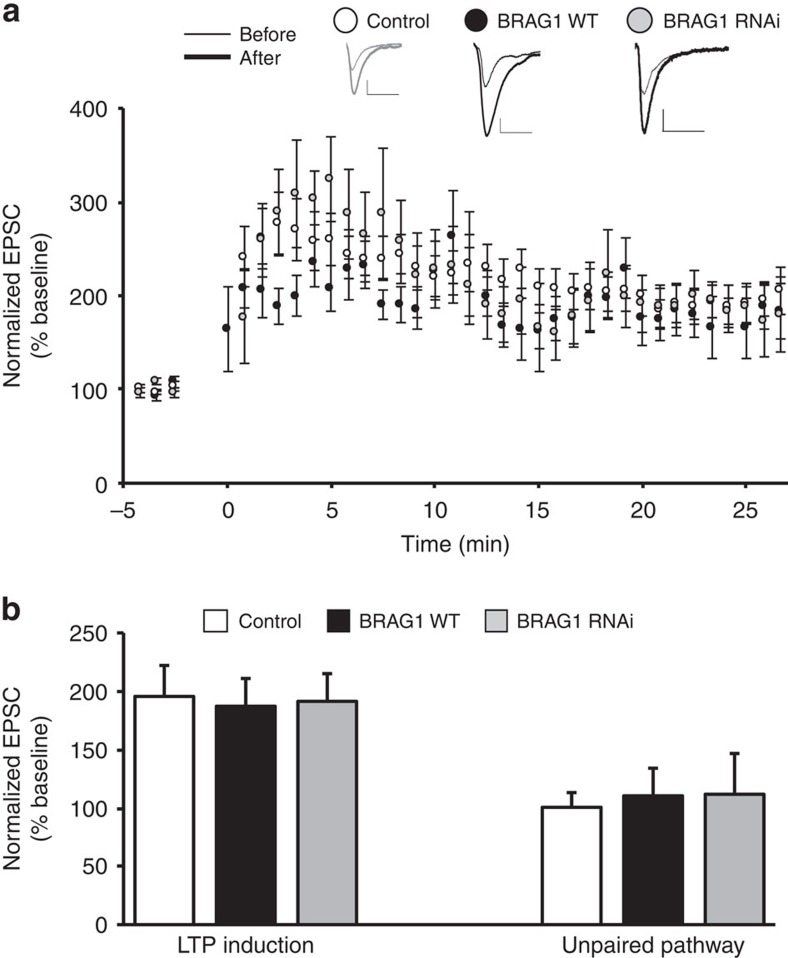
BRAG1 is not required for LTP. (**a**) LTP was induced by pairing 3 Hz presynaptic stimulation (300 pulses) with 0 mV postsynaptic depolarization (indicated by an arrow) in CA1 neurons while expressing BRAG1 (black circles, *n*=7, *P*=0.96), BRAG1 RNAi (grey circles, *n*=5, *P*=0.86) or untransfected neurons (white circles, *n*=19). Insets: sample traces of evoked AMPAR-mediate responses recorded at −60 mV before pairing (thin line) and 20 min after pairing (thick line) from control or transfected cells as indicated. Scale bars, 20 pA, 20 ms. (**b**) Normalized averaged steady-state AMPAR-mediated responses in paired (LTP induction) and control (unpaired pathway).

At excitatory synapses in the CA1 region of the hippocampus, LTP is mediated by AMPAR insertion and is dependent on the activation of NMDARs. To determine whether BRAG1-mediated enhancement of synaptic transmission blocks activity-dependent insertion of AMPARs, we investigated the effect of BRAG1 expression on LTP. LTP was induced in BRAG1-transfected and untransfected CA1 neurons by pairing presynaptic stimulation (3 Hz, 1.5 min) with postsynaptic depolarization (0 mV). As shown in Fig. 9, BRAG1 expression did not interfere with LTP expression, indicating that activity-independent BRAG1-mediated enhancement of synaptic transmission does not occlude activity-dependent AMPAR insertion. To further analyse the role of BRAG1 in LTP, we tested the effect of the RNAi on LTP. As shown in Fig. 9, knocking down BRAG1 does not interfere with LTP, further supporting the conclusion that BRAG1 is not required for LTP expression.

### Arf-GEF activity of BRAG1 is required for LTD

NMDA receptor-dependent LTD is characterized by the activity-dependent removal of AMPARs. To directly test whether BRAG1 Arf-GEF activity is required for the activity-dependent removal of synaptic AMPARs, we tested whether BRAG1-E849K expression interferes with NMDA receptor-dependent LTD. LTD was induced in BRAG1-E849K-transfected and untransfected CA1 neurons by pairing presynaptic stimulation (1 Hz, 8.3 min) with postsynaptic depolarization (−40 mV).

**Figure 10:**
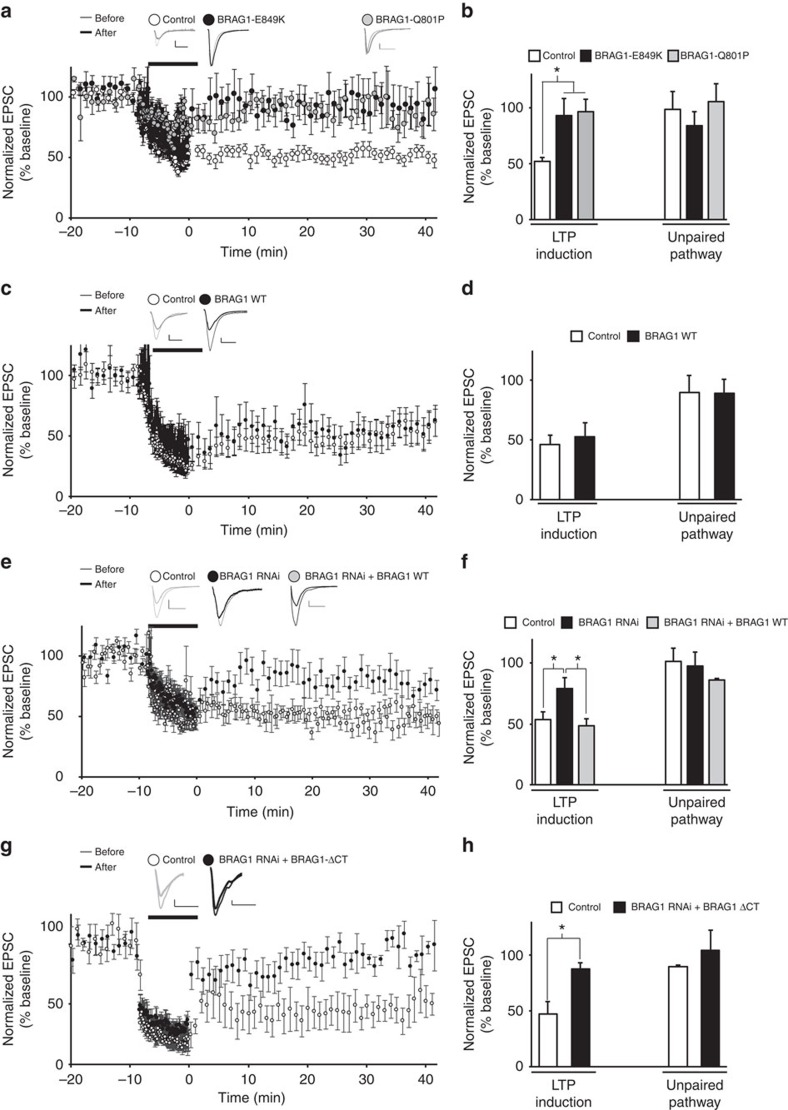
Arf-GEF activity and PDZ-binding sequence are required for LTD. LTD was induced by pairing a low-frequency stimulation (500 pulses at 1 Hz) with −40 mV postsynaptic depolarization (indicated by a dark line) in untransfected CA1 neurons or neurons transfected with BRAG1-E849K (**a**; *n*=9), BRAG1-Q801P (**a**; *n*=8), wild-type BRAG1 (**c**; *n*=7), BRAG1 RNAi (**e**; *n*=7), BRAG1 RNAi+wild-type BRAG1 (**e**; *n*=9) or BRAG1 RNAi+BRAG1-ΔCt (**g**; *n*=6). Insets: sample traces of evoked AMPAR-mediated synaptic responses recorded at −60 mV before pairing (thin line) and 20 min after pairing (thick line) from control or transfected cells as indicated. Scale bars, 20 pA, 20 ms. (**b**,**d**,**f**,**h**) Normalized average steady-state AMPAR-mediated responses in paired (LTD induction) and control (unpaired pathway). * Indicates significance (*P*≤0.05).

This protocol produces a ∼50% decrease in the EPSCs from the baseline in control (untransfected) cells. In contrast, cells expressing E849K failed to exhibit LTD (Fig. 8a,b), supporting the critical role of Arf-GEF activity of BRAG1 in the activity-dependent removal of AMPARs. The inset in Fig. 8a is a representative sample of simultaneous paired recordings, which reveals enhanced baseline EPSCs in BRAG1-E849K cells (consistent with Fig. 1b), and that this enhancement persists despite LTD induction. To test the role of XLID mutant (BRAG1-Q801P) in LTD, we tested the effect of BRAG1-Q801P expression on LTD. As shown in Fig. 10a, and similar to BRAG1-E848K, BRAG1-Q801P blocked LTD. These data indicate that the Arf-GEF activity of BRAG1 is required for LTD in CA1 hippocampal neurons. As a control, we tested the effect of wild-type BRAG1 expression on LTD induction. As shown in Fig. 10c, cells expressing BRAG1 exhibited LTD similar to their control untransfected cells.

To further analyse the role of BRAG1 in LTD, we tested the effect of knocking down BRAG1 in LTD. As shown in Fig. 10e, cells expressing BRAG1 RNAi were not able to express LTD. Importantly, BRAG1 co-expression with the RNAi was able to rescue LTD.

To analyse the role of the PDZ-binding sequence in LTD expression, we co-expressed the RNAi with the BRAG1-ΔCt. Interestingly, BRAG1-ΔCt was not able to rescue LTD in cells expressing the RNAi (Fig. 10g), indicating the importance of the PDZ-binding sequence in BRAG1 function in LTD.

Taken together, these data indicate that while the PDZ-binding sequence of BRAG1 is required for the two major functions of BRAG1, maintenance of synaptic transmission and LTD, the Arf-GEF activity is only required for LTD.

## Discussion

A recent study showed that four different mutations in BRAG1 cause XLID. Each of the four mutations (R359C, R758Q, Q801P and R863W) results in decreased BRAG1 Arf-GEF activity^1^. In this study, we explored the synaptic function of BRAG1. First, we show that the Arf-GEF activity of BRAG1 is required for the maintenance of a major form of synaptic plasticity, namely, LTD, suggesting a possible mechanism underlying cognitive deficits in these families with BRAG1 XLID. While investigating the role of the Arf-GEF enzymatic activity of BRAG1 (as the common effect of each of the mutations linked to XLID) in synaptic function, we discovered that BRAG1 also bidirectionally regulates synaptic transmission. Thus, this study highlights a novel dual role of BRAG1 in synaptic function.

The bidirectional role of BRAG1 in synaptic transmission was evident through combining overexpression and knockdown and molecular replacement approaches. While decreasing the levels of BRAG1 reduces synaptic transmission, increasing its levels enhances synaptic transmission. Our data present a novel role of BRAG1 as a rate-limiting molecule in increasing synaptic transmission in an activity-independent manner. This conclusion is supported by several experimental observations. (1) BRAG1 expression increases basal synaptic transmission in the form of AMPAR-mediated responses without affecting NMDAR-mediated responses, GABA receptor-mediated responses or PPF. (2) The BRAG1-mediated increase in synaptic transmission is independent of neuronal activity (as evident by the increased synaptic transmission in the presence of TTX). (3) It is also independent of NMDAR activation. (4) BRAG1-mediated enhancement in synaptic transmission is also independent of BRAG1's Arf-GEF activity, as both the XLID mutant (BRAG1-Q801P), which has reduced enzymatic activity, and the BRAG1-E849K mutant, which lacks enzymatic activity, increase basal synaptic transmission. (5) BRAG1 enhances transmission independent of GluA1 insertion as demonstrated in the rectification experiment. (6) BRAG1 increases the surface expression of the GluA2 subunit but not the GluA1 subunit as evident by the use of super ecliptic PHlourin-tagged subunits. (7) Finally, Pep2m (the peptide that interferes with the GluA2/3 recycling pool) abolished the difference in synaptic transmission between BRAG1-expressing and control neurons. Interestingly, while BRAG1-mediated enhancement of synaptic GluA2/3 receptors is independent of its enzymatic activity, it does require its PDZ-binding motif.

The BRAG1-mediated increase in synaptic transmission could be attributed to one or more of the following: (1) increase in AMPAR insertion; (2) reduction in AMPAR removal; (3) increase in synapse number; and (4) increase in AMPAR conductance. Our data that BRAG1 increases surface expression of GluA2, along with the peptide data strongly suggest that BRAG1 enhances synaptic transmission, at least partly, through increased insertion of AMPARs. Given the important role of BRAG1 in the activity-dependent removal of AMPARs, it is unlikely that the overexpression of BRAG1 would reduce the AMPAR removal. However, this possibility, along with whether BRAG1 plays a role in synapse number and AMPAR conductance, requires further studies to be fully addressed.

BRAG1 possesses two different motifs (PDZ-binding motif and a sec 7 domain) that may mark BRAG1 as a critical link between scaffolding molecules (for example, BRAG1 binds PSD-95 (ref. 49)) and small GTPases (for example, BRAG1 activates Arf6 (refs 4, 49)). In addition to the Arf-GEF-dependent removal of AMPARs, our data show that BRAG1 functions as a rate-limiting molecule in the enhancement of synaptic AMPARs in an activity-independent manner. Taken together, BRAG1 may be critical in the assembly and/or trafficking of a slot protein complex.

In 2001, Shi *et al*. proposed that ‘slot complexes' might encode synaptic strength by acting as placeholders for AMPARs. Once slots are created, they will be filled by GluA2/3 receptors, creating a new and higher baseline for synaptic transmission^50^,^51^. The composition of this slot protein complex is largely unknown; however, the ability of BRAG1 to increase synaptic AMPARs in a manner that requires PDZ interaction, and that is independent of activity suggests that BRAG1 may facilitate the organization of the slot protein complex, possibly in a rate-limiting manner.

Although BRAG1 does not interact directly with GluA2 (ref. 52), the PDZ-binding motif is necessary for BRAG1-mediated enhancement of GluA2 responses. BRAG1 does, however, interact with PDZ domains of several members of the MAGUK family including PSD-95 (ref. 49), a proposed slot protein^53^. Like BRAG1, PSD-95 does not directly bind AMPARs, yet it is necessary and sufficient to enhance synaptic transmission through its interactions with stargazin^54^,^55^,^56^. However, unlike BRAG1, PSD-95 regulates activity-dependent GluA1 insertion without affecting the recycling GluA2 (ref. 57). One possible mechanism for selectively creating slots for GluA2/3 receptors is that following activity-dependent delivery of PSD-95/stargazin/GluA1/2 complexes to the synapse, BRAG1 binds to PSD-95, positioning itself to both remove synaptic GluA1/2 receptors through Arf-GEF activity and also to create a ‘slot' for GluA2/3 replacing GluA1/2. Thus, BRAG1 may act as a ‘molecular memory' for synaptic AMPAR number^58^,^59^. A potential protein that may be also involved in this slot complex is S-SCAM (synaptic scaffolding molecule; also called membrane-associated guanylate kinase inverted-2), a PSD protein that contains six PDZ domains and whose expression, such as BRAG1, increases synaptic GluA2/3 receptors. Whether BRAG1 and S-SCAM interact to regulate synaptic GluA2/3 receptors needs further exploration. Interestingly, the BRAG family member BRAG3, which also includes a C-terminal sequence for binding class I PDZ domains, directly interacts with S-SCAM through its WW domain^60^. We find the possible role of BRAG1 as a ‘slot complex organizer' directly orchestrating the replacement of GluA1 with constitutively recycling GluA2 receptors to be an intriguing possibility requiring a great deal of further exploration.

Another important question that remains to be answered is the role of BRAG1 in NMDAR function. RNAi experiments show that knockdown of BRAG1 results in a reduction in NMDAR-mediated responses. These effects could be secondary to the effects of BRAG1 on AMPARs. However, replacement experiments show that the BRAG1 that lacks the PDZ-binding sequence is unable to rescue AMPAR-mediated responses but rescues NMDAR-mediated responses. This suggests that BRAG1 may be important in NMDAR function, independent of its ability to bind to PDZ proteins. The possible role of BRAG1 in NMDAR function is also independent of its enzymatic function as the replacement experiments show that BRAG1-Q801P mutant is capable of rescuing NMDAR-mediated responses as well. Further studies are warranted to explore the potential role of BRAG1 in NMDAR function.

The regulatory effect of BRAG1 Arf-GEF activity on AMPAR removal was made evident by the inability of neurons expressing the BRAG1-E849K or the BRAG1-Q801P mutants to express LTD. Furthermore, knockdown of BRAG1 clearly indicates the important role of BRAG1 in LTD expression. In support of a role of the Arf-GEF activity of BRAG1 in AMPAR removal, a recent study showed that the Arf-GEF activity of BRAG1 is required for JNK-mediated removal of AMPARs^61^. An interesting finding in this study is that the PDZ-binding motif of BRAG1 is required not only for the maintenance of basal synaptic transmission but also for the activity-dependent removal of AMPARs (LTD). It is possible that the BRAG1's PDZ-binding sequence switches binding partners that contains PDZ motifs and thus regulates the stability and the removal of AMPARs. A possible candidate that BRAG1 may be interacting with through its PDZ-binding sequence is PICK1 (protein that interacts with C kinase 1), which is known to be required for the expression of LTD^62^. Further studies are needed to address these hypotheses.

Our data provide strong evidence that BRAG1 expression enhances synaptic transmission through AMPAR-mediated responses that are independent of BRAG1's enzymatic activity. These results, however, are in contrast with a recent publication, by Myers *et al*.^61^, which found that BRAG1 expression results in depressed AMPAR-mediated responses^61^. We cannot fully explain the reason for this discrepancy. A possible explanation for the disparity over BRAG1's effect on synaptic transmission could be due to the age difference in the hippocampal slices used for electrophysiological experiments. While Myers *et al*.^61^ performed experiments on hippocampal slices that remained in culture for 7–14 days, our protocol used slices that were cultured for 3–8 days. It is worth noting, however, that our analysis of >10 different electrophysiological experiments revealed that the BRAG1-mediated increase in synaptic transmission is independent of the age of the slices (in the time frame used; Supplementary Fig. 3).

An important aspect of this study is the clinical relevance for BRAG1 in XLID. Activation of Arf6 by BRAG1 is impaired by each of the identified XLID-linked mutations. Our finding that the Arf-GEF activity of BRAG1 is required for NMDAR-dependent LTD suggests that this feature of synaptic plasticity may be misregulated when BRAG1 is mutated. Interestingly, the importance of normal LTD is also illustrated in Fragile X syndrome, another X-linked cognitive disorder. Metabotropic GluR-dependent LTD is excessively expressed in the mouse model for Fragile X syndrome^63^,^64^,^65^,^66^. Michalon *et al*. (2012) showed that CTEP, a selective mGlu5 inhibitor, was able to correct both the enhanced mGluR-LTD and the cognitive deficits and auditory hyperactivity in the Fragile X mouse model^71^. This reinforces the importance of synaptic plasticity balance in cognitive function, and suggests that misregulation of this important aspect of synaptic plasticity may contribute to the neurological deficits caused by BRAG1 mutations. Thus, it is clear that additional studies are warranted to further understand the role of BRAG1 in higher-order cognitive processes.

## Methods

### Animals and slice cultures

Organotypic hippocampal slice cultures were prepared from postnatal day 5 or 6 Sprague-Dawley rats of either sex (Charles River laboratories, Portage, MI, USA; Harlan laboratories, Madison, WI, USA) as described previously^45^,^67^. All biosafety procedures and animal care protocols were approved by the Medical College of Wisconsin Institutional Animal Care and Use Committee. DNA constructs were introduced to cells following 2–7 days in culture using the biolistic (gene gun) delivery method as described^68^,^69^. All constructs were expressed overnight, except where specified. All chemicals were obtained from Sigma (St Louis, MO, USA) except where noted.

### DNA constructs and expression

FLAG-tagged human wild-type BRAG1 (BRAG1) and BRAG1-E849K in the pCAGGS vector were generated as described previously^4^. Truncated FLAG-BRAG1 with a C-terminal truncation (FLAG-BRAG1-ΔCt) was amplified by PCR from the KIAA0522 template (kindly provided by the Kazusa DNA Research Institute, Chiba, Japan) with sense primer 5′- TTAACCTCTAGAAATGGATTACAAGGAT GACGACGATAAGGAGAGAGCGGGGACAGGG -3′ to incorporate an N-terminal FLAG-tag and XbaI site and antisense primer 5′- CGGCTTGGCTCGAGCTAGGGGTTTGCAC -3′ to incorporate and XhoI site and point mutation (underlined) to generate a premature stop codon to delete the nine C-terminal amino acids. The PCR product was cloned into pCAGGS and the presence of the mutation was verified by sequencing.

Enhanced GFP (EGFP)-BRAG1 fusion proteins were generated by recloning pCAGGS-BRAG1 and mutant constructs into pEGFP-C1. BRAG1 sequences were removed from pCAGGS vectors and inserted into pEGFP vectors downstream of EGFP along with linker DNA containing restriction enzyme sites that allow insertion into the correct open reading frame. Custom oligos were ordered from Invitrogen (linker 1: 5′- GTACATTCTAGACTA CTAGTTGTAC -3′, linker 2: 5′- TCGAGTACAACTAGTAGTCTAGAAT -3′) and annealed to create a double-stranded segment of DNA containing XbaI and SpeI restriction enzyme cut sites surrounded by 5′ and 3′ single-stranded overhangs compatible to overhangs resulting from digestion with BsrG1 and XhoI, respectively. The single-stranded oligos were annealed in buffer #3 (New England Biolabs, Ipswich, MA, USA) for 4 min at 95 °C, 10 min at 70 °C and for 60 min at room temperature. After annealing the oligos, DNA Ligation Kit Version 1 (Clontech Laboratories, Inc, Mountain View, CA, USA) was used to ligate the linker DNA into pEGFP-C1 that had been digested with BsrG1 (New England Biolabs) and XhoI (Invitrogen, Waltham, MA, USA). Ligated DNA was transformed into Max Efficiency DH5α Competent Cells (Invitrogen). Correct insertion of the linker DNA was verified via a screening digest with the SpeI enzyme (Invitrogen), the restriction site for which was present in plasmids that contained the linker DNA but not in the original pEGFP-C1 vector. The resulting vector, pEGFP linker, contained XhoI and XbaI restriction enzyme sites appropriate for insertion of BRAG1 digested from pCAGGS-BRAG1. Ligation and transformation were carried out using the reagents mentioned above and successful cloning was verified via a screening digest with BsrG1, which would cut the plasmid three times if it contained the BRAG1 insertion but cut the original vector only once. This same approach was used to clone the BRAG1 mutants into pEGFP linker.

### RNA interference

Three unique 19-nucleotide sequences targeting rat BRAG1 were designed and the pSuper RNAi system was used. This system uses a mammalian expression vector that directs intracellular synthesis of siRNA-like transcripts. The sequences designed are: BRAG1 RNAi1: 5′- GCCAGTATCGTATGAATAA -3′, BRAG1 RNAi2: 5′- GCATCCAAGGTCGTGAACT -3′, BRAG1 RNAi3: 5′- GGCTACGCTTTACCTCTGA -3′.

### Electrophysiology

Double whole-cell patch-clamp evoked responses were recorded simultaneously from adjacent transfected and untransfected CA1 pyramidal neurons, with bipolar stimulating electrodes placed on Schaffer collateral fibres as described previously^45^, using Multiclamp 700A amplifier (Axon Instruments). All recordings were performed in circulating artificial cerebral spinal fluid (ACSF) composed of 119 mM NaCl, 2.5 mM KCl, 4 mM CaCl_2_, 4 mM MgCl_2_, 26 mM NaHCO_3_, 1 mM NaH_2_PO_4_ and 11 mM glucose at pH 7.4 bubbled in 5% CO_2_, 95% O_2_. Recording pipettes (3–6 mΩ) were filled with internal solution containing 115 mM cesium methanesulfonate, 20 mM CsCl, 10 mM HEPES, 2.5 mM MgCl_2_, 4 mM Na_2_ATP, 0.4 mM Na_3_GTP, 10 mM sodium phosphocreatine and 0.6 mM EGTA, at pH 7.25. Picrotoxin (0.1 μM; Acros Organics, NJ, USA) and 2-chloroadenosine (2 μM) were added to the ACSF for all recordings except miniature inhibitory postsynaptic currents (IPSC's). ACSF for recording miniature IPSCs, measuring GABA receptor-mediated current, included 0.1 μM APV (R&D Systems, MN, USA), 10 μM CNQX and 1 μM TTX (Tocris Bioscience, Bristol, UK).

LTP was induced by stimulating presynaptic fibres with 300 pulses at 3 Hz frequency (in the paired pathway) while depolarizing the cell to 0 mV (control pathway received no stimulation during induction). Baseline (2.5 min) and post-induction responses were evoked with 0.2 Hz from alternating electrodes (pathways). LTD was induced with a stimulation frequency of 500 pulses at 1 Hz while cells were held at −40 mV (control pathway receives no stimulation during induction). Baseline and post-induction periods were stimulated at 0.1 Hz.

Rectification was accomplished by introducing spermine (0.1 μM final concentration; Acros Organics, NJ, USA) to internal solution, and APV (0.1 μM final concentration) to the ACSF. For peptide experiments shown in Fig. 4, internal solution filled with 10 μM pep2m (Tocris Biosciences, Bristol, UK) was infused into cells through the patching glass micropipettes and responses were evoked at 0.2 Hz and measured simultaneously from untransfected and BRAG1-transfected cells. Cell-permeable peptides (used in Fig. 2d,e) contained either the last nine amino acids of BRAG1 C-term (KPSRISTVV) or its corresponding control (KPSRISAVA) with an additional HIV-derived TAT sequence (GRKKRRQRRR) and a fluorescein amidite tag (488 nm) to visualize peptide localization. The peptides were custom made at the Blood Research Institute Protein Core lab at the Medical College of Wisconsin. Peptides are synthesized as peptide acids and purified using reverse phase high-performance liquid chromatography.

### Confocal imaging

All imaging experiments were performed with a Leica TCS SP5 confocal microscope. The processing and the analysis of acquired images were carried out with ImageJ. Imaging experiments in Figs 1 and 6 were carried out with live organotypic hippocampal slices in ACSF gassed with 5% CO_2_, 95% O_2_ and maintained at 37 °C in a live-imaging chamber. SEP-GluA constructs were expressed via biolistic transfection along with td-tomato and either pCAGGS-BRAG1 or pCAGGS as a control. pCI-SEP-GluR1 and pCI-SEP-GluR2(R) were gifts from Robert Malinow (Addgene plasmids #2,4000 and #2,4001, respectively). Spine-to-dendrite ratios were measured as previously described^45^,^46^,^70^. Briefly, fluorescence intensity across a section of dendrite, spine and adjacent background was quantified. Background fluorescence was subtracted from the spine and its adjacent dendrite. Fluorescence intensity of spine-to-dendrite ratio of SEP-GluA was normalized to that of td-tomato. Imaging for Fig. 7 was carried out on slices fixed with 4% formaldehyde/4% sucrose and stained with anti-BRAG1 antibody.

### Protein overlay assay

Membranes with HEK293 cell lysates overexpressing WT BRAG1 or BRAG1-ΔCt and no plasmid or with GST-PSD-95 and GST were incubated with 10 μg ml^−1^ GST-PSD-95 (diluted in TBST) or with 20 μg ml^−1^ of HEK293 cell lysates overexpressing WT BRAG1 or BRAG1-ΔCt (diluted in TBST+5% Triton X-100), respectively, overnight at room temperature. The membranes were washed in TBST, stained and prepared for immunoblotting. Membranes were stained with BRAG1 antiserum C3 (1:10,000), PSD-95 antisera UCT80 (1:10,000), anti-FLAG (1:1000; Cat# F3165, Sigma, St Louis, MO, USA), or anti-tubulin (1:10,000; Cat# ab11316, Abcam, Cambridge, MA, USA). The membranes were then incubated with horseradish peroxidase-conjugated secondary antibodies (Jackson Immunoresearch Laboratories, West Grove, PA, USA) for chemiluminescent detection with West Pico substrate (Pierce, Rockford, IL, USA). To reprobe the membranes with a different primary antibody, bound antibodies were stripped from the membrane with Restore reagent (Pierce).

### Statistical analysis

Evoked paired recordings were analysed with a paired non-parametric Wilcoxon test. When more than two conditions were compared, one-way analysis of variance was used. Unpaired Student's *t*-test was used to analyse all other experiments. Shapiro–Wilk test was used to test for normality. Error bars denote the s.e. of the mean in all cases.

## Additional information

**How to cite this article:** Brown, J. C. *et al*. Bidirectional regulation of synaptic transmission by BRAG1/*IQSEC2* and its requirement in long-term depression. *Nat. Commun.* 7:11080 doi: 10.1038/ncomms11080 (2016).

## Supplementary Material

Supplementary Information Supplementary Figures 1-7
