# Supplementary material for: Bidirectional regulation of synaptic transmission by BRAG1/IQSEC2 and its requirement in long-term depression
Source: Nat Commun. 2016 Mar 24;7:11080. doi: 10.1038/ncomms11080 (PMC4820844; doi:10.1038/ncomms11080)
Supplement: Supplementary Information — Supplementary Figures 1-7 [file ncomms11080-s1.pdf]

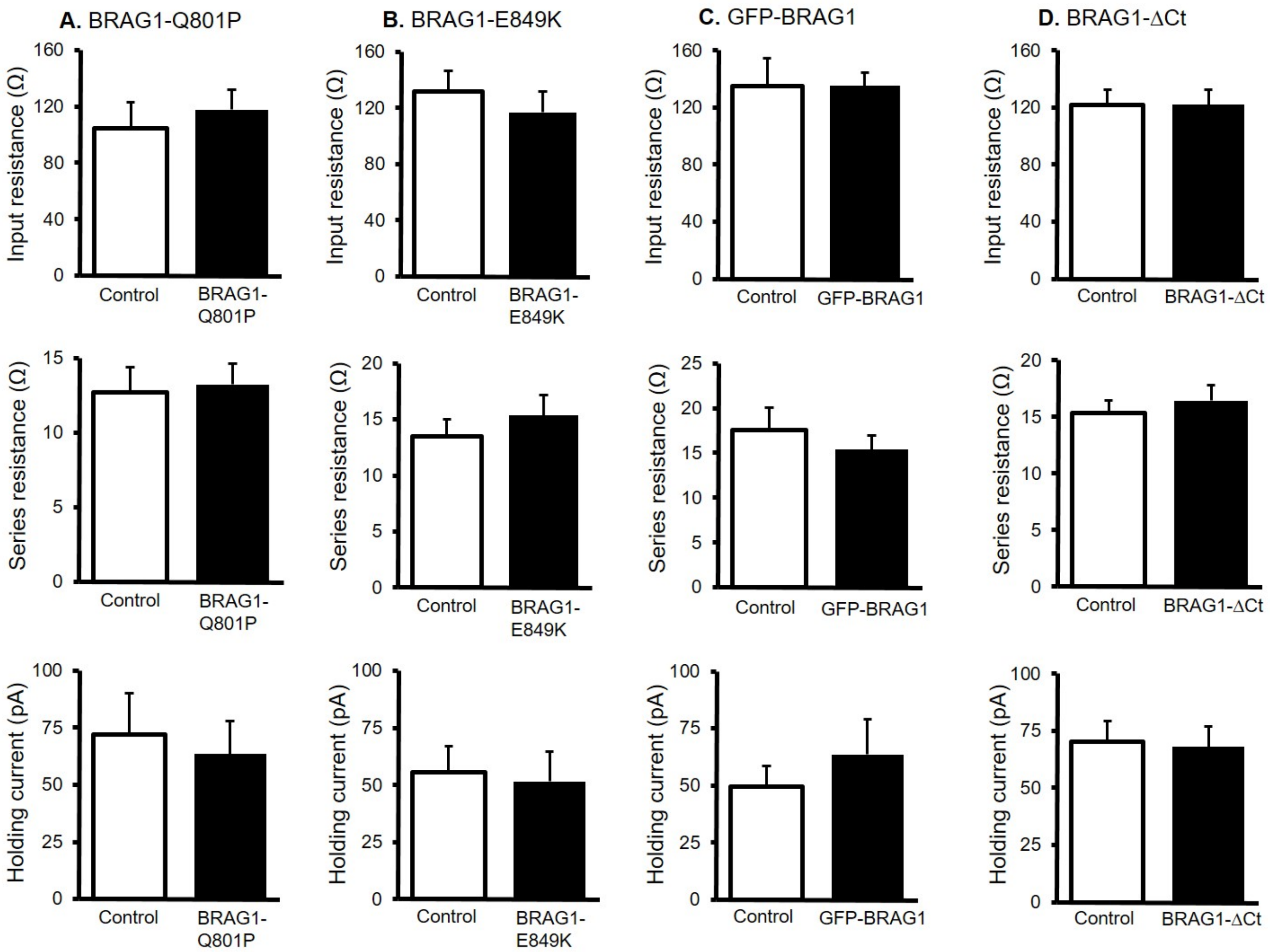

Supplementary Figure 1. The effect of the expression of BRAG1 and its mutants on cell input resistance (IR), series resistance (SR) and holding current (HC). Data represent average input resistance (*top panel*), series resistance (*middle panel*), and holding current (*bottom panel*) from pairs of untransfected cells and their corresponding transfected cells expressing BRAG1-Q801P (**A**) (n = 11; IR: p = 0.34; SR: p = 0.55; HC: p = 0.61), BRAG1-E849K (**B**) (n = 14; IR: p = 0.31; SR: p = 0.56; HC: p = 0.69), GFP-BRAG1 (**C**) (n = 8; IR: p = 0.21; SR: p = 0.69; HC: p = 0.46), or BRAG1-ΔCt (**D**) (n = 19; IR: p = 0.99; SR: p = 0.17; HC: p = 0.82). Pairs were excluded if holding current was less than -150 pA or if series resistance differed between pairs by two-fold or more. Error bars represent standard error of the mean.

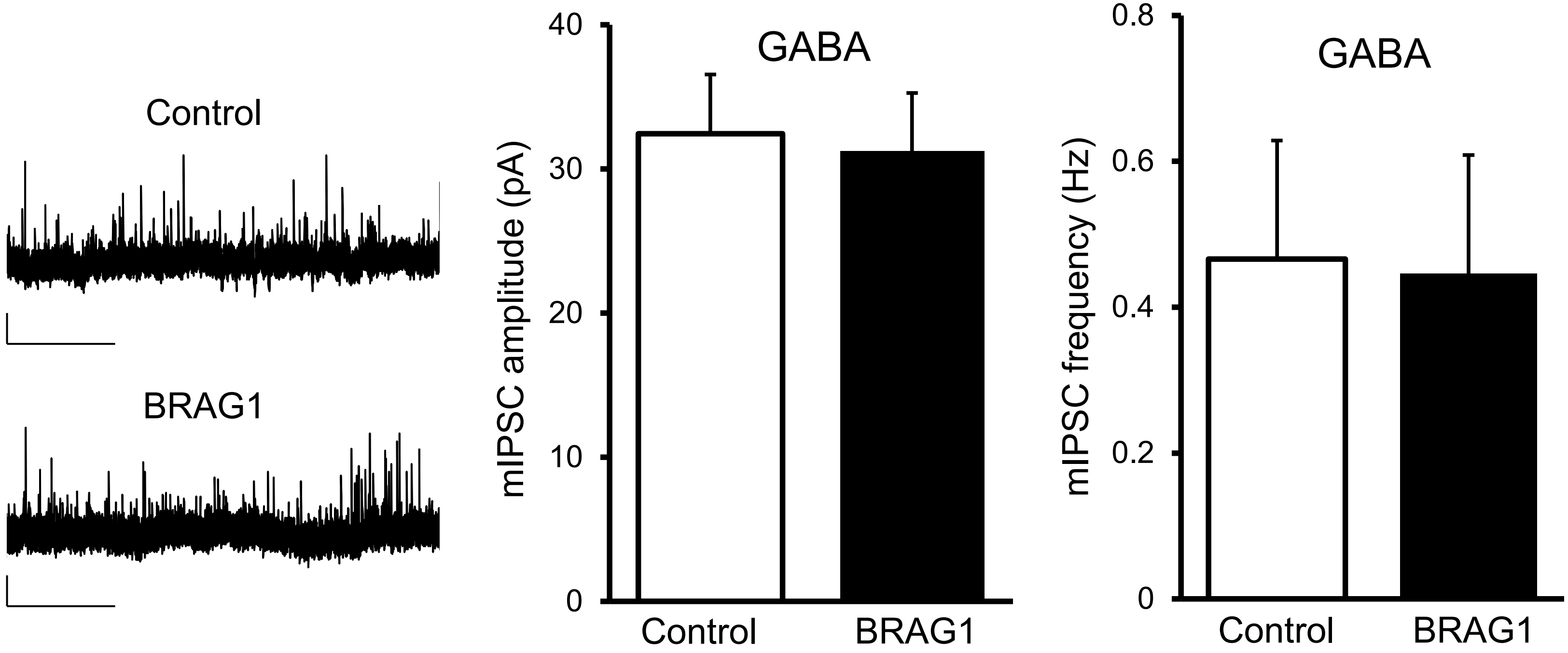

Supplementary Figure 2. BRAG1 does not alter GABA receptor-mediated transmission.
Insets, sample traces of GABA receptor-mediated mIPSCs recorded at 0 mV with 0.1 μM APV and 10 μM CNQX in ACSF. Scale bar 20pA, 10 sec. Bar graphs represent averaged amplitude of GABA receptor-mediated mIPSC (*left*, control: n = 14; BRAG1: n = 13; p = 0.83) and mIPSC frequency (*right*, control: n = 14; BRAG1: n = 13; p = 0.93). Error bars represent standard error of the mean.

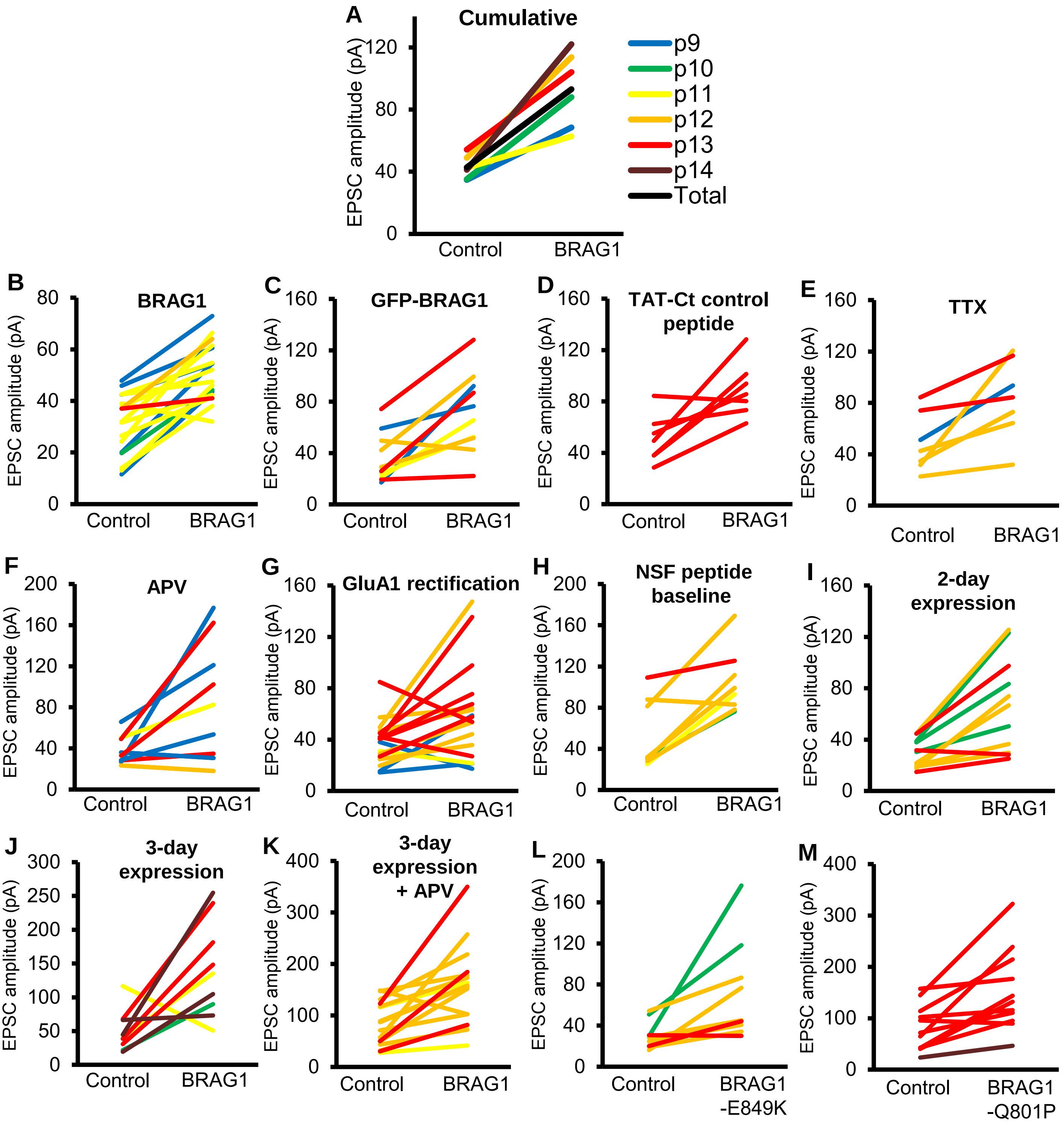

Supplementary Figure 3. Lack of correlation between age of the neurons and AMPAR-mediated transmission. Graphs demonstrate relationship of neuron age at time of recording, including *in vitro* culture time (ranging from postnatal day P9 to P14). Colored lines represent pair recordings of control neurons (*left*) and neurons transfected with BRAG1 (*right*). (**A**) Composite data from all experiments performed with overexpression of BRAG1 and BRAG1 mutants. (**B-M**) Raw data of pair recordings are represented by a line colored according to age of tissue at time of recording.

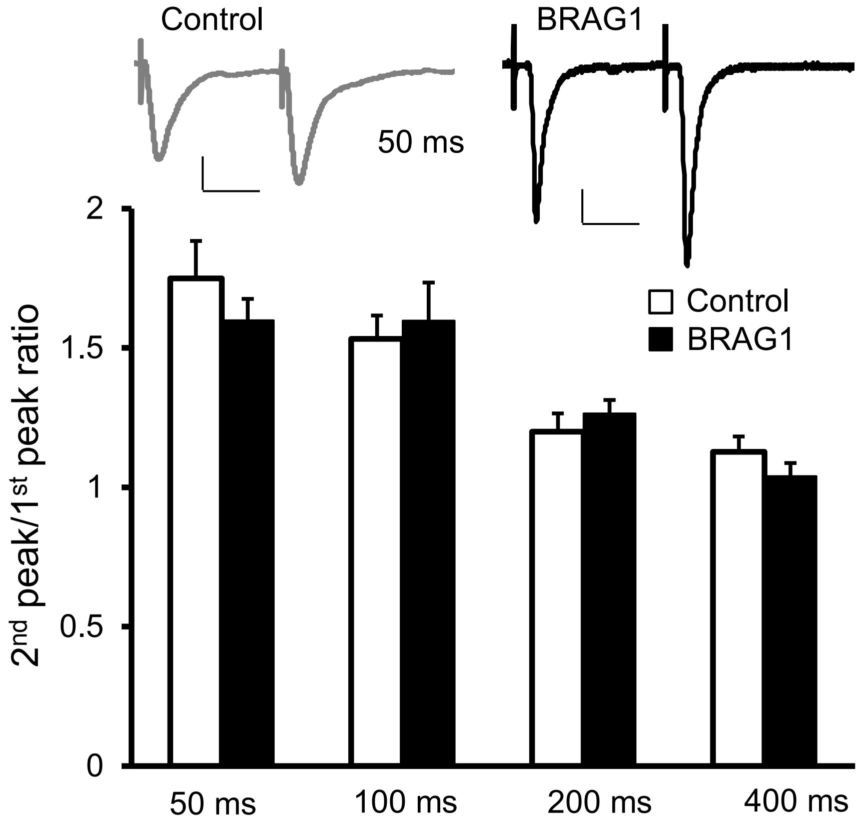

Supplementary Figure 4. Paired-pulse facilitation is not changed by BRAG1. *Inset,* paired-pulse facilitation (PPF) sample traces of initial evoked response, followed by a second evoked response after 50 ms. Data represent average PPF ratios obtained by dividing the second (latent) peak (recorded at intervals of 50 ms, 100 ms, 200 ms, and 400 ms for each cell) by the first (initial) peak. (*50 ms*: control: n = 14; BRAG1: n = 14; p = 0.35; *100 ms*: control n = 14; BRAG1: n = 14; p = 0.68; *200 ms*: control: n = 13; BRAG1: n = 13; p = 0.43; *400 ms*: control: n = 13; BRAG1: n = 13; p = 0.24). *Scale bars,* 20 pA, 20 ms. Error bars represent standard error of the mean.

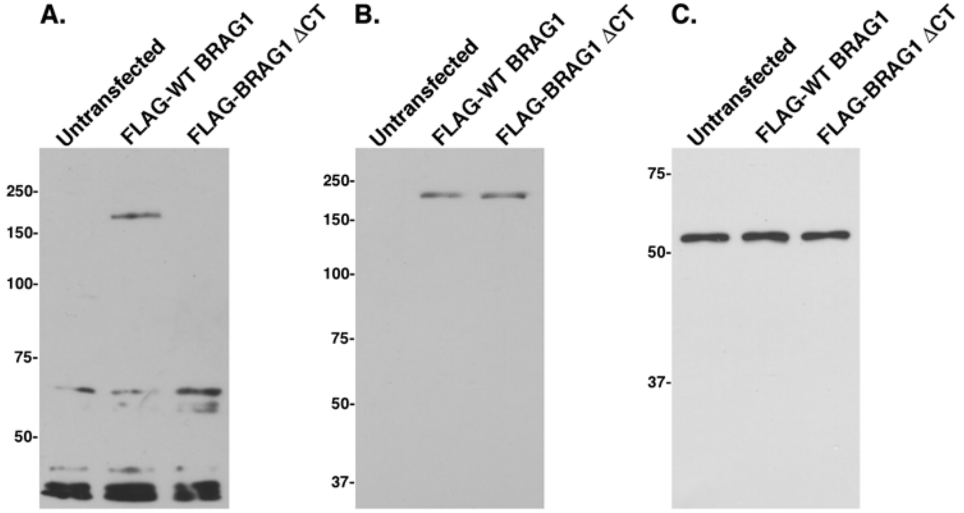

Supplementary Figure 5: Full length immunoblots demonstrating that the C-terminal region of BRAG1 is necessary for binding to PSD-95 *in vitro*. Blots were prepared as described in Figure 2A. A. PSD-95 was overlaid on a blot of cell lysates expressing FLAG-WT BRAG1 or FLAG-BRAG1 C-terminal deletion mutant (FLAG-BRAG1 ΔCT), and the blot was stained against PSD-95. The PSD-95 bound FLAG-WT BRAG1 but not FLAG-BRAG1 ΔCT. B. Full length blot showing FLAG-WT BRAG1 and FLAG-BRAG1 Δ CT expression. C. The cell lysates were stained against tubulin as a loading control.

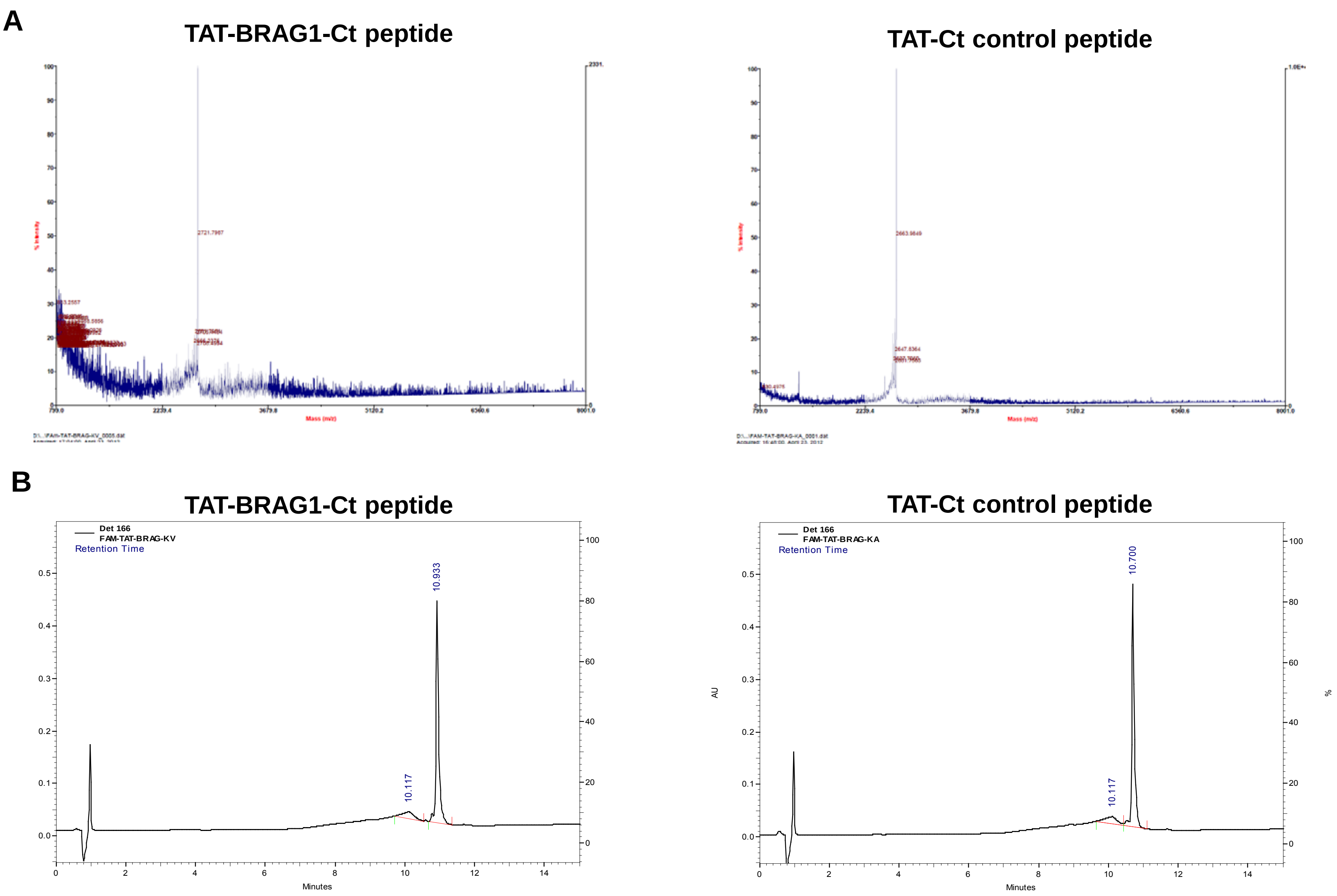

Supplementary Figure 6. Analysis of identity and purity of BRAG1 peptides. (**A**) Mass spectral analysis (ESI-MS and MALDI-TOF) of the BRAG1 PDZ-interfering peptide (KPSRISTVV) and its control peptide (KPSRISTAA). (**B**) RP-HPLC elution profiles of BRAG1 peptides. The peptides were RP-HPLC purified using a Proteo C-12 column and a 0.1%TFA/ACN gradient and lyophilized.

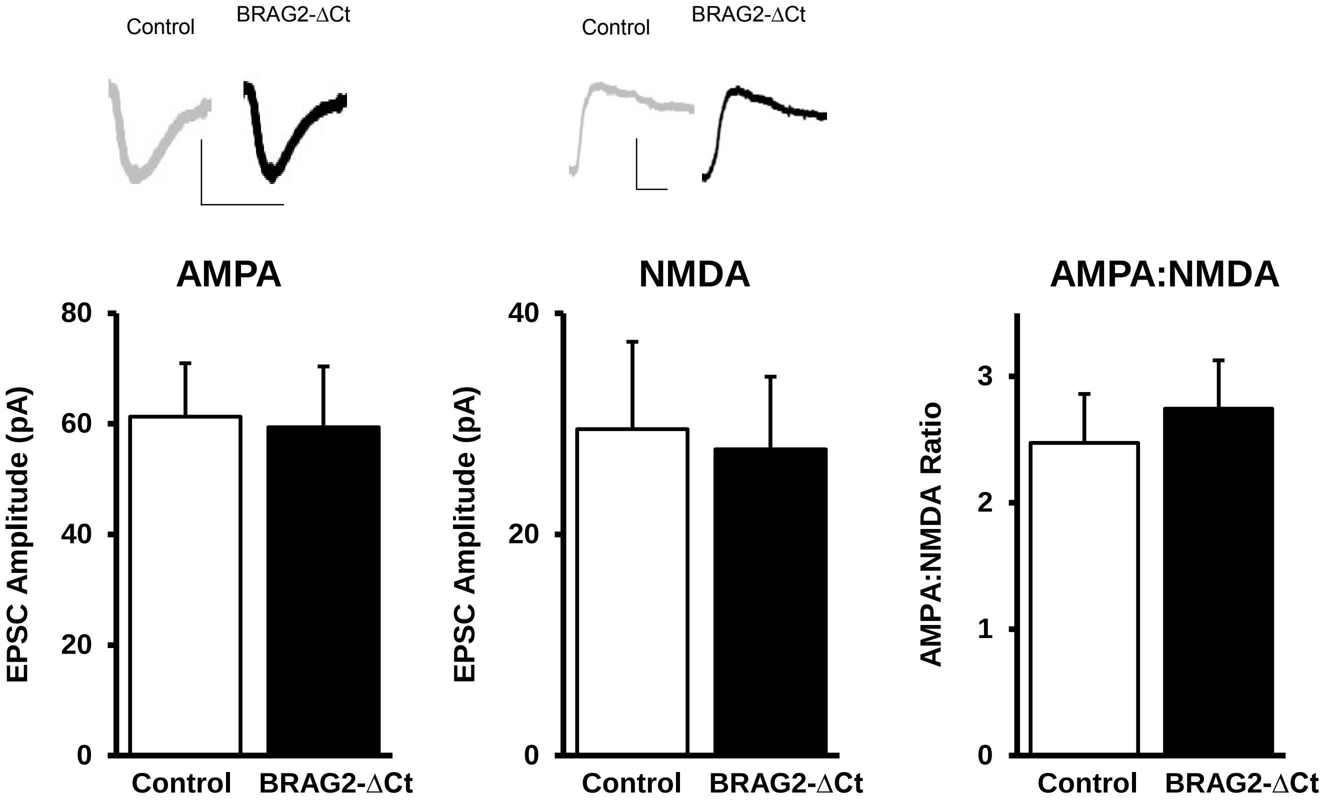

Supplementary Figure 7. BRAG2 lacking the PDZ-binding sequence has no effect on basal synaptic transmission. *Left panels,* sample traces of AMPAR- and NMDAR-mediated responses recorded at -60 mV and +40 mv, respectively. *Scale bars,* 20 pA, 20 ms. Data represent averaged evoked EPSCs recorded for AMPA (*left graphs*), NMDA (*center graphs*), and AMPA/NMDA ratios (*right graphs*) simultaneously from pairs of untransfected (control) CA1 neurons and neurons transfected with BRAG2-ΔCt (AMPA: $n = 11$, $p = 0.90$; NMDA: $n = 7$, $p = 0.86$; AMPA/NMDA: $n = 7$, $p = 0.42$).
